# Applications of Artificial Intelligence in Endobronchial Ultrasound for Lung Cancer Diagnosis and Staging: A Scoping Review

**DOI:** 10.3390/curroncol33050287

**Published:** 2026-05-13

**Authors:** Jacobo Echeverri-Hoyos, Jaime A. Echeverri-Franco, Nicole Bonilla, Gustavo Monsalve-Morales, Eduardo Tuta-Quintero

**Affiliations:** 1 School of Medicine, Institución Universitaria Visión de las Américas, Pereira 660000, Colombia; jacoboecheverri9@gmail.com; 2 Pulmonology, Clínica de Alta Tecnología Oncólogos del Occidente, Pereira 660000, Colombia; jaime.echeverrif@gmail.com; 3 School of Medicine, Universidad de La Sabana, Chía 250001, Colombia; nicolebosi@unisabana.edu.co; 4 School of Medicine, Universidad Tecnológica de Pereira, Pereira 660000, Colombia; gustavomonsalvem@gmail.com; 5 Department of Epidemiology and Internal Medicine, Universidad de La Sabana, Chía 250001, Colombia

**Keywords:** lung cancer, diagnosis, endobronchial ultrasound, artificial intelligence

## Abstract

**Introduction**: Lung cancer remains highly lethal. Endobronchial ultrasound (EBUS) enables minimally invasive diagnosis and staging. Artificial intelligence (AI) improves image analysis and diagnostic accuracy, though current evidence is limited by retrospective, small, single center studies. **Methods**: A scoping review following Arksey–O’Malley, Levac, and JBI frameworks, was reported as per PRISMA-ScR. Databases were searched for studies (2015–2026) on AI in EBUS. Two reviewers screened, extracted standardized data, and performed narrative synthesis grouped by algorithm type, application, and performance metrics. **Results**: A total of 26 studies were included. Of these, 73.1% (19/26) employed deep learning-based models, while 26.9% (7/26) used traditional or hybrid machine learning approaches. The most frequent clinical objective was diagnostic classification of malignancy (14/26; 53.8%), followed by segmentation or cytological analysis (5/26; 19.2%), anatomical navigation or lymph node station classification (3/26; 11.5%), and multimodal predictive or staging support models (4/26; 15.4%). Most studies were based on EBUS-derived images or videos (18/26; 69.2%), including both convex-probe and radial-probe applications. Studies were distributed among Convex Probe-EBUS for mediastinal staging, Radial Probe-EBUS for peripheral lesion assessment, and rapid on-site evaluation-based cytology analysis, reflecting diverse clinical contexts. Most models were developed using static images. **Conclusions**: AI applications in EBUS are predominantly based on deep learning and mainly focused on diagnostic classification, with growing but still limited exploration of segmentation, navigation, and multimodal approaches. The evidence reflects diverse clinical contexts and data sources, particularly image-based inputs, but remains unevenly distributed across applications.

## Simple Summary

Lung cancer is one of the leading causes of cancer-related death, and early and accurate diagnosis is essential to improve patient outcomes. Endobronchial ultrasound is a minimally invasive procedure used to examine and sample lung lesions and lymph nodes. In recent years, artificial intelligence has been applied to improve the interpretation of these images. In this review, we analyzed current research on artificial intelligence in this setting. Most studies show promising results, particularly for identifying cancer and analyzing images, but they vary widely in quality and methods. Many are based on small datasets and lack validation in real-world conditions. While some systems can assist doctors in real time, they are not yet reliable enough to replace clinical judgment. Future research should focus on larger, standardized studies to ensure these technologies can be safely and effectively used in routine clinical practice.

## 1. Introduction

Lung cancer (LC) remains one of the leading causes of mortality worldwide and represents a major clinical challenge due to its high incidence and the frequent diagnosis at advanced stages [1,2]. The identification of parenchymal lesions, whether central or peripheral, as well as mediastinal lymph node involvement, determines critical therapeutic decisions such as surgery, chemotherapy, radiotherapy, or immunotherapy [3,4]. Therefore, its evaluation using minimally invasive diagnostic methods constitutes a fundamental component of the clinical approach [3,4,5].

Endobronchial ultrasound-guided bronchoscopy (EBUS) is a widely used diagnostic modality in interventional pulmonology for obtaining biopsies in patients with suspected neoplastic disease or other mediastinal pathologies, while also enabling mediastinal lymph node staging [6,7]. Its diagnostic yield depends on multiple variables, including operator experience, the use of rapid on-site evaluation (ROSE), the use of different sampling tools during the procedure, the number of lymph node stations sampled, ultrasonographic features suggestive of malignancy, and the size and location of the lesion [6,7,8].

The incorporation of artificial intelligence (AI)-based tools has emerged as a promising strategy to optimize diagnostic accuracy by detecting complex patterns not identifiable through human inspection, facilitating tasks such as classification, segmentation, and clinical prediction [9,10]. Chen et al. developed AI-CEMA, an AI system for automated analysis of Convex Probe-EBUS videos that identifies and classifies benign and malignant lymph nodes. Trained on 1006 lymph nodes and validated in 267 multicenter cases, it achieved an AUC of 0.84, with performance comparable to that of clinical experts [11]. Similarly, Yong et al. employed a deep convolutional neural network based on a modified VGG16 architecture for the automatic classification of malignant thoracic lymph nodes in endobronchial ultrasound-guided transbronchial needle aspiration (EBUS-TBNA) images; using 2394 samples, the model achieved an accuracy of 75.8%, sensitivity of 72.7%, specificity of 79.0%, and an AUC of 0.80 [12].

Despite these advances, the current literature presents important limitations. Most available studies are retrospective, single-center, and based on small sample sizes, which limits their clinical generalizability [9,10,11,12,13]. Therefore, it is necessary to systematically map the current development of AI applied to EBUS, identify its main areas of application, and recognize the methodological gaps that must be addressed prior to large-scale clinical adoption. The aim of this study is to synthesize the available evidence on the applications of AI in EBUS-based bronchoscopy, evaluating its diagnostic performance, clinical applicability, and the methodological limitations of published studies.

## 2. Methods

### 2.1. Study Design

A scoping review of the literature was conducted following the methodological framework proposed by Arksey and O’Malley [14] and further refined by Levac [15], as well as the methodological guidance from the Joanna Briggs Institute [16] for scoping reviews. This scoping review was conducted and reported in accordance with the PRISMA Extension for Scoping Reviews (Preferred Reporting Items for Systematic Reviews and Meta-Analyses extension for Scoping Reviews, PRISMA-ScR) [17] (Supplementary File S1), with the aim of mapping and synthesizing the available evidence on the application of AI in the diagnosis and staging of LC using EBUS. This scoping review was not prospectively registered.

### 2.2. Research Question

The research question was formulated using the PCC framework (Population, Concept, and Context). The population included adult patients with suspected or confirmed LC; the concept referred to the use of artificial intelligence, including machine learning, deep learning (DL), and computer-aided diagnostic systems; and the context focused on the application of EBUS as a diagnostic or mediastinal staging tool. Based on this framework, the following research question was defined: What evidence exists regarding the use of AI for the diagnosis and staging of LC using EBUS?

### 2.3. Eligibility Criteria

Original observational studies or clinical trials published between 2015 and 2026 in English or Spanish were included. Eligible studies involved adult patients with suspected or confirmed LC, evaluated AI tools applied to EBUS-derived images, and reported diagnostic performance metrics such as sensitivity, specificity, accuracy, or area under the curve (AUC). Narrative reviews, systematic reviews, letters to the editor, abstracts without full results, studies lacking diagnostic performance data, and publications in which AI was applied to LC without a direct relationship to EBUS were excluded.

### 2.4. Search Strategy

The literature search was conducted in the electronic databases PubMed, Scopus, and Embase, selected for their comprehensive coverage of biomedical sciences and medical technology. Combinations of controlled vocabulary and keywords related to artificial intelligence, LC, and EBUS were used, applying Boolean operators (AND, OR) to maximize search sensitivity (Supplementary File S2).

### 2.5. Study Selection

**Figure 1:**
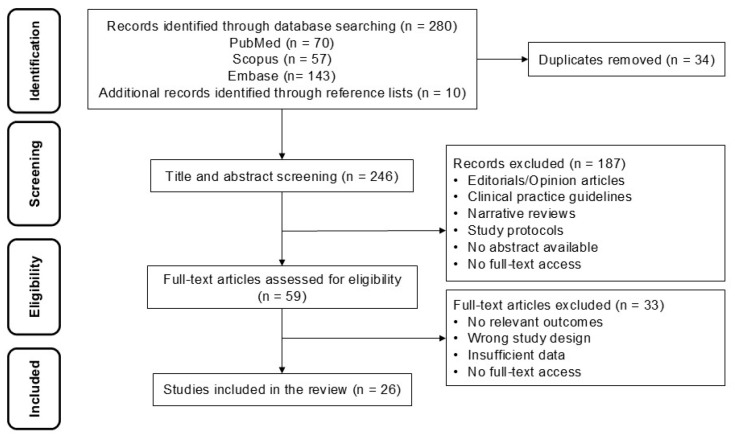
PRISMA-ScR flowchart.

The web-based application Rayyan was used for the independent screening of candidate publication abstracts, followed by discussion and consensus for study inclusion by two reviewers [18]. The selection process was carried out in two phases. In the first phase, duplicate records retrieved from different databases were removed, followed by screening of titles and abstracts to identify potentially eligible studies. In the second phase, full-text articles were assessed to confirm compliance with the inclusion criteria. Study selection was performed systematically, with discrepancies resolved through consensus. The study selection process is presented in a PRISMA-ScR flow diagram (Figure 1).

### 2.6. Data Extraction and Synthesis of Results

Data extraction was performed using a predesigned standardized matrix, in which variables such as author/year, AI type/model, clinical objective, clinical application, sample size, main outcomes, and limitations were recorded. This process enabled the homogeneous organization of the information to facilitate comparison across studies.

Results are presented through a narrative synthesis and comparative tables, grouping studies according to the type of algorithm used, the clinical purpose of AI (diagnosis or staging), and the reported performance metrics. The findings of this scoping review are reported based on the categories proposed by Grudniewicz et al. [19]: (i) a summary of the characteristics and distribution of the included studies, and (ii) a narrative synthesis of the results. This approach will allow the identification of methodological trends, strengths of current models, and relevant knowledge gaps for future research in the field.

The heterogeneity of the included studies was further explored through a structured framework applied during the results synthesis. Studies were categorized across four pre-defined dimensions: imaging modality (Convex Probe-EBUS, Radial Probe-EBUS, or ROSE/cytology), input data type (static images, video-based, or multimodal), validation strategy (internal, external, or multicenter), and clinical objective (diagnosis, staging, navigation, or segmentation/cytology). This table provides representative examples of studies within each category; all included studies were considered in the overall synthesis.

## 3. Results

A total of 26 studies were included (Figure 1). Of these, 73.1% (19/26) employed deep learning-based models, while 26.9% (7/26) used traditional or hybrid machine learning approaches. The most frequent clinical objective was diagnostic classification of malignancy (14/26; 53.8%), followed by segmentation or cytological analysis (5/26; 19.2%), anatomical navigation or lymph node station classification (3/26; 11.5%), and multimodal predictive or staging-support models (4/26; 15.4%). Most studies were based on EBUS-derived images or videos (18/26; 69.2%), including both convex-probe and radial-probe applications. A subset of studies incorporated multimodal data—combining EBUS with computed tomography, positron emission tomography, or clinical variables (6/26; 23.1%)—while others focused on ROSE-based cytological imaging (3/26; 11.5%) (Table 1).

Ervik et al. (2024) [20] developed a U-Net-based model for automatic segmentation of lymph nodes and vessels, achieving Dice coefficients of 0.71 and 0.76, respectively, with near real-time processing. In a subsequent study, Ervik et al. (2026) [21] applied DenseNet-121 with Grad-CAM for lymph node station classification, achieving moderate accuracy (63.1%) and demonstrating clinical interpretability through attention maps.

Chen CH et al. (2019) [22] implemented a hybrid CNN-SVM model that achieved an accuracy of 85.4% and an AUC of 0.87, outperforming traditional methods. Similarly, Lin CK et al. (2025) [13] proposed a Transformer-based multimodal model (TransEBUS), achieving an accuracy of 82% and an AUC of 0.88, with performance comparable to experienced clinicians. Xing et al. (2024) [26] reported outstanding results using an optimized FKNN model, achieving an accuracy of 99.38%, although with potential overfitting due to the limited sample size. Likewise, Ishiwata et al. (2024) [29] achieved diagnostic metrics close to 96.7% across all variables using SqueezeNet.

Yong et al. (2022) [12] reported an accuracy of 75.8% using a modified VGG16 model in real-time settings. Hotta et al. (2022) [36] achieved high sensitivity (95.3%) but low specificity (53.4%), limiting its utility for confirmatory diagnosis. Similarly, Patel et al. (2024) [38] reported high specificity (96.9%) but low sensitivity (43.2%), suggesting primary utility for ruling in malignancy. Guberina et al. (2022) [24] integrated computed tomography/positron emission tomography data with machine learning models, achieving an AUC > 0.93 and high sensitivity (~94.5%). More recently, Oh et al. (2025) [35] developed a multimodal model (EBUS + computed tomography/positron emission tomography + computed tomography), achieving an AUROC of 0.914, demonstrating the value of integrating multiple data sources.

Regarding segmentation and cytology, Lan et al. (2024) [28] achieved outstanding performance (F1 = 0.96) in ROSE images, while Wang et al. (2022) [37] reported high accuracy (93.4%) in whole-slide segmentation. Chen et al. (2025) [11] developed the AI-CEMA system, achieving an AUC of 0.889 (retrospective) and 0.849 (prospective), high sensitivity (97.1%), and real-time processing capability. In contrast, Amante et al. (2025) [40] reported a balanced accuracy of 71.5% in Iriscope videos, outperforming junior physicians but not expert clinicians.

Structured heterogeneity analysis (Table 2) revealed substantial variability across multiple dimensions of AI applications in EBUS. Studies were distributed among Convex Probe-EBUS for mediastinal staging, Radial Probe-EBUS for peripheral lesion assessment, and ROSE-based cytology analysis, reflecting diverse clinical contexts. Most models were developed using static images; however, a growing number of studies incorporated video-based data and multimodal approaches integrating images with clinical or radiological variables. Validation strategies showed a predominance of internal validation methods, with relatively few studies performing external or multicenter validation, highlighting limitations in generalizability. Clinical objectives varied widely, with diagnostic classification being the most frequent application, followed by staging, segmentation/cytology, and anatomical navigation.

## 4. Discussion

This scoping review demonstrates a growing application of AI in interventional pulmonology using EBUS for the diagnosis and staging of LC. The predominance of deep learning-based models reflects a clear trend toward the use of advanced architectures, particularly convolutional neural networks, for image analysis in bronchoscopy, where automated image processing has shown advantages. The most frequently identified application was diagnostic classification of malignancy, highlighting the clinical interest in improving the characterization of lymph nodes and pulmonary lesions during EBUS procedures. However, variability in performance across studies indicates that these systems are not yet fully robust or generalizable. Overall, this multidimensional heterogeneity underscores the lack of standardization among studies and represents a key challenge for the comparison and clinical translation of AI models in EBUS.

Applications in segmentation and cytological analysis demonstrated high performance, as shown by Lan et al. [28], where an F1-score of 0.96 was achieved. These applications appear to be more mature, possibly due to more structured tasks with lower clinical variability compared to diagnostic classification. In contrast, applications in anatomical navigation (12.5%), such as those proposed by Ervik et al. [20,21], highlight the potential of AI to assist in real-time during procedures, although with lower performance compared to segmentation and cytological analysis tasks.

One of the most relevant findings is the high heterogeneity in diagnostic performance [12,26,29]. This variability likely reflects differences in sample size, data quality, class balance, and validation strategies. Additionally, these studies revealed an imbalance between sensitivity and specificity, indicating that many models are primarily optimized for a single function: either detecting cases (high sensitivity) or confirming them (high specificity) [36,37,38]. Multimodal models, which integrate EBUS with other data sources such as computed tomography, positron emission tomography, or clinical variables, may improve diagnostic accuracy. This suggests that the future of AI in EBUS will likely not rely solely on endobronchial imaging, but rather on models that combine multiple modalities [24,35].

Several studies reported exceptionally high diagnostic performance, such as the 99.38% accuracy achieved with the FKNN-based model and values close to 96–97% with SqueezeNet [26,29]. While these results are promising, they should be interpreted with caution. Both studies were conducted with relatively small, single-center datasets and lacked external validation, which substantially increases the risk of overfitting and limits the generalizability of the findings. Furthermore, the use of highly selected or balanced datasets can further inflate performance estimates by reducing real-world variability.

An analysis of this variability suggests that the differences in diagnostic performance reflect structural heterogeneity among studies. There is substantial variation in the composition of the datasets, including differences in image acquisition protocols, annotation strategies, and class distribution. Studies that rely on highly selected datasets or frame-level selection tend to report higher performance, while those using full video sequences or real-world clinical data show more modest and variable results, as seen in studies using prospective or video-based datasets [11,13,40]. Conversely, studies with smaller or highly selected datasets often report very high-performance metrics [26,29,33], suggesting that some of the reported accuracy may be due to data selection bias rather than true model generalizability.

Furthermore, the wide range of reported performance metrics may be partially explained by differences in validation strategies. Several studies used internal cross-validation or randomized splits without external validation [22,25,39], which could overestimate performance. Studies incorporating independent or multicenter datasets demonstrated a consistent decline in performance, highlighting the impact of domain switching [11,34]. This suggests that model robustness remains a significant limitation and that current results should be interpreted with caution when extrapolating to different clinical settings.

Real-time processing capability is essential for implementation in guided procedures [11,20]. AI-CEMA demonstrated solid performance with prospective validation and adequate processing speed, positioning it as one of the models closest to clinical application [11,20]. However, although AI may outperform less experienced operators, it has not yet reached the level of expert clinicians. This reinforces the notion that, in the short term, AI should be considered as a supportive tool rather than a replacement for clinical judgment or expertise [11,20,40].

The observed imbalance between sensitivity and specificity in the different models also reflects differences in optimization goals and clinical approach. Models developed on datasets enriched with malignant cases or trained with detection-prioritizing loss functions tend to achieve high sensitivity at the expense of specificity [11,36]. Models that achieve high specificity often show limited sensitivity, favoring confirmatory use over screening [23,27,38]. This divergence suggests that AI models in EBUS are not converging toward a single “optimal” diagnostic tool but rather are evolving into task-specific systems with distinct clinical functions, which has important implications for their integration into clinical workflows or clinical decision-making.

The improved performance observed in multimodal models further underscores a key limitation of image-only approaches. EBUS images provide localized structural information but lack broader anatomical and metabolic context. By integrating computed tomography, positron emission tomography, or clinical variables, multimodal models reduce uncertainty and improve discrimination, as demonstrated by studies combining imaging modalities and clinical features [24,30,35]. This suggests that the variability observed in unimodal models may be partly due to intrinsic limitations of the data modality itself, rather than algorithmic shortcomings. Consequently, future research should focus less on incremental architectural improvements and more on data integration strategies and the fusion of clinically relevant features.

According to the available medical evidence, including recent systematic and narrative reviews [10,43,44] there is general agreement regarding the predominance of deep learning-based models and their high diagnostic potential in the context of EBUS and bronchoscopy. However, our review highlights greater heterogeneity in study designs, sample sizes, and performance metrics. It also identifies important limitations, such as the imbalance between sensitivity and specificity, limited external validation, and the predominance of retrospective single-center studies. Taken together, these findings underscore a significant gap between performance observed in controlled settings and effective implementation in real-world clinical practice.

The variability between studies is not only a methodological problem but also reflects a field that is still in an exploratory phase, lacking standardized benchmarks, with heterogeneous outcome definitions and limited reproducibility. This is reinforced by the predominance of single-center retrospective designs and the scarcity of cross-study external validation [11,25,34,39]. Therefore, emphasis must be placed on external validation, the diversity of datasets, and clinically relevant endpoints. Without these elements, the risk remains that AI systems may perform well under controlled conditions but fail to deliver consistent benefit in routine clinical practice.

Beyond methodological limitations, regulatory approval remains a significant challenge, as most AI models have not undergone prospective validation or evaluation within formal regulatory frameworks [45]. Furthermore, variability in data acquisition protocols across centers limits model generalizability and complicates standardization [46]. Integration into bronchoscopy workflows also presents practical challenges, particularly regarding real-time processing requirements, user interface design, and seamless interaction with existing clinical systems [47]. Finally, hardware limitations and computational demands may restrict the implementation of advanced models in routine settings [48]. Successful clinical translation of AI in EBUS will depend on addressing these technical, regulatory, and workflow-related challenges [45,46,47,48].

From a clinical perspective, these tools are more likely to be integrated as real-time decision support systems during EBUS procedures, assisting in lymph node characterization, guiding sampling, or providing anatomical orientation [11,12,13,20], rather than replacing clinical judgment. AI can offer the greatest benefit as a complement for less experienced operators, helping to standardize interpretation and reduce variability between operators [36,40], while expert bronchoscopists can use these systems as complementary tools to increase diagnostic confidence [11,31,40].

### Limitations and Strengths

As a scoping review, no formal assessment of methodological quality or risk of bias of the included studies was performed, as the primary objective was to map the available literature rather than quantitatively synthesize results. Consequently, no meta-analysis or standardized comparison of performance metrics across studies was conducted. However, the review protocol and methodological framework were registered and made publicly available through the Open Science Framework (OSF) [49], representing an important methodological strength by enhancing the transparency, reproducibility, and credibility of the review process.

The inclusion of highly heterogeneous studies in terms of design, population, AI models, and outcome variables may limit the comparative interpretation of findings. Another important limitation is the lack of consistent adherence to established reporting guidelines for AI in medical research, which may compromise transparency, reproducibility, and comparability across studies [50,51,52]. The absence of standardized reporting frameworks further limits the ability to adequately assess methodological rigor and to reliably interpret differences in model performance.

Overall, the quality of the evidence base is limited by the predominance of retrospective, single-center studies, with only a minority including external validation, which is essential to assess model robustness across different clinical settings. Additionally, many studies reported small sample sizes or highly selected datasets, which may increase the risk of overfitting and overestimation of performance. The heterogeneity in reported metrics and validation methods further complicates direct comparisons between studies and limits the ability to draw definitive conclusions. Although the search strategy was comprehensive, it was restricted to selected databases and publications in English and Spanish, potentially excluding relevant evidence published in other languages or non-indexed sources.

## 5. Conclusions

The application of AI in EBUS for LC diagnosis and staging is characterized by a clear predominance of deep learning-based approaches, primarily focused on diagnostic classification, with emerging roles in segmentation, navigation, and multimodal integration. Real-time processing capability and the integration of multiple data sources represent important advances. However, the current evidence base remains highly heterogeneous in terms of study design, data sources, and performance metrics, and is largely dominated by retrospective, single-center studies with limited external validation.

## Figures and Tables

**Table 1:** Applications of artificial intelligence in endobronchial ultrasound for lung cancer diagnosis and staging.

Author (Year)	AI Type/Model	Clinical Objective	Clinical Application	Sample	Main Results	Limitations
Øyvind Ervik et al. (2024) [20]	U-Net (convolutional neural network for segmentation)	Automatic segmentation of mediastinal lymph nodes and blood vessels in EBUS images	Real-time assistance during EBUS-TBNA for anatomical identification and bronchoscopist support	1161 images from 40 patients (1307 lymph nodes and 800 vessels annotated; 134 images in test set)	Simultaneous segmentation with Dice scores of 0.71 (lymph nodes) and 0.76 (vessels); detection rate of 98% for lymph nodes and 64% for vessels; near real-time processing (~55 ms/image), supporting clinical feasibility	Small sample size, single-center study, lower performance for vessels, and slight limitations for optimal real-time speed
Øyvind Ervik et al. (2026) [21]	CNN (DenseNet-121) + Grad-CAM (explainable AI)	Classification of mediastinal lymph node stations in EBUS images	Automated anatomical navigation with model explainability to support bronchoscopists and training	35,527 images from 75 patients (3131 images in test set)	Overall accuracy of 63.1% (precision 62.8%, sensitivity 59.0%, F1-score 59.1%); Grad-CAM demonstrated that model attention frequently overlapped with clinically relevant anatomical structures; substantial inter-observer agreement in interpretability assessment (81.6%, kappa 0.529)	Moderate classification performance limits immediate clinical adoption, variability across lymph node stations, and reliance on expert interpretation for validation of explainability
Chen CH et al. (2019) [22]	CNN (fine-tuned CaffeNet) + SVM (hybrid model with transfer learning)	Differentiation between benign and malignant pulmonary lesions using EBUS images	Intraoperative diagnostic support for lesion characterization	164 cases (56 benign, 108 malignant; evaluated with 5-fold cross-validation)	Hybrid CNN-SVM model achieved accuracy 85.4%, sensitivity 87.0%, specificity 82.1%, and AUC 0.87; significantly outperformed CNN alone and handcrafted feature-based methods; transfer learning and asymmetric data augmentation improved performance	Limited dataset size, class imbalance requiring augmentation strategies, and lack of external validation
Patel YS et al. (2024) [23]	NeuralSeg (deep learning model for segmentation and elastography analysis)	Assessment of lymph node malignancy using EBUS elastography	Selection of suspicious lymph nodes prior to biopsy (EBUS-TBNA guidance)	187 lymph nodes (from 124 patients; prospective single-center study)	Diagnostic accuracy 70.6%, sensitivity 43.0%, specificity 90.7%, PPV 77.3%, NPV 68.5%, and AUC 0.82; AI-based stiffness area ratio (SAR) and CLNS were significant predictors of malignancy, supporting its role as a decision-support tool	Low sensitivity limiting detection of malignant nodes, single-center design, and dependence on predefined elastography thresholds
Lin CK et al. (2025) [13]	Hybrid Transformer (TransEBUS: CNN + Transformer + two-stream multimodal + MoCo)	Classification of benign vs. malignant mediastinal lesions from EBUS-TBNA videos	Automated multimodal EBUS video analysis integrating grayscale, Doppler, and elastography for diagnostic support	330 EBUS videos from 150 patients (50 lesions in independent test set)	Accuracy 82%, sensitivity 84.2%, specificity 80.7%, and AUC 0.88; outperformed conventional CNN/3D models and achieved performance comparable to experienced clinicians; demonstrated real-time capability (~0.19 s per clip) and effective multimodal feature integration	Single-center dataset, lack of external validation, and potential overfitting due to limited data and homogeneous acquisition settings
M. Guberina et al. (2022) [24]	Random Forest + MLP (with comparison to logistic regression)	Prediction of lymph node metastasis (EBUS positivity) using PET/CT and clinical features	Complementary tool to PET/CT for radiotherapy planning and nodal staging	675 lymph node stations from 180 patients with stage III NSCLC	High sensitivity (94.5%) comparable to expert readers; AUC > 0.93 for all models (MLP best); lower misclassification rates than standard PET/CT assessment; combined models further improved sensitivity (~96–97%) while reducing false negatives	Restricted to stage III NSCLC population, single-center dataset, and performance dependent on feature engineering (PET/CT-derived variables)
H. Wang et al. (2025) [25]	Multi-branch deep learning framework (ensemble of CNNs with voting and optimization strategies)	Classification of peripheral pulmonary lesions (benign vs. malignant) using Radial Probe-EBUS images	Computer-aided diagnosis for peripheral bronchoscopy (Radial Probe-EBUS-guided procedures)	95 patients with Radial Probe-EBUS videos (multi-frame image dataset derived from videos; 4-fold cross-validation)	Best performance achieved with AUC 0.80, accuracy 0.78, sensitivity 85%, and specificity 72%; multi-branch ensemble with Bayesian optimization outperformed single-branch and prior methods, improving robustness to class imbalance	Single-center study, limited sample size, lack of external validation, and challenges in model interpretability
J. Xing et al. (2024) [26]	Optimized Fuzzy KNN with feature selection (bECMRFO-FKNN)	Prediction of lung cancer malignancy using R-EBUS, CT, and clinical-biochemical features	Decision support tool for lung cancer diagnosis integrating multimodal clinical data	156 patients undergoing R-EBUS-guided biopsy	Very high performance: accuracy 99.38%, sensitivity 100%, specificity 98.89%, and MCC 98.82%; outperformed classical ML models and other feature selection approaches; robust feature selection enhanced discrimination	Single-center retrospective dataset, relatively small sample size, potential overfitting due to extremely high performance, lack of external validation
Y. Ito et al. (2022) [27]	CNN (Xception-based AI-CAD)	Prediction of lymph node metastasis using EBUS-TBNA ultrasound images	Support for lymph node selection and diagnostic assessment during EBUS-TBNA	91 patients, 166 lymph nodes (6444 images)	High specificity (90.2% CV; 95.0% hold-out) and good overall accuracy (87.9% hold-out), but low sensitivity in cross-validation (37.3%); better performance in hold-out (sensitivity 76.9%), indicating usefulness for confirming metastasis	Moderate sample size, variability between validation methods, low sensitivity in cross-validation, single-center study
S. H. Yong et al. (2022) [12]	CNN (modified VGG16 with GAP and custom loss function)	Classification of malignant lymph nodes in EBUS images	Real-time assistance during EBUS-TBNA	2394 images from 888 lymph nodes (310 patients)	Moderate accuracy (75.8%), sensitivity 72.7%, specificity 79.0%, and AUC 0.80; real-time performance (~63 images/second) with improved results over standard architectures	Intermediate performance, reliance on manual lymph node annotation, single-center study, variability across model configurations
H. Lan et al. (2024) [28]	CUNet3+ (fully convolutional network)	Cytological cell segmentation in ROSE-stained images	Automation of rapid cytopathological analysis during EBUS procedures	130 ROSE cytology images (augmented to >50,000 patches)	Excellent segmentation performance (F1 = 0.9604, Dice = 0.9150); high accuracy (0.9834); outperformed cytopathologists and demonstrated fast inference (~0.116 s/image); maintained strong performance in external validation (F1 ≈ 0.91)	Small original dataset, heavy reliance on data augmentation, predominantly single-center training data, evaluation partly based on cropped/selected images
T. Ishiwata et al. (2024) [29]	CNN (SqueezeNet, transfer learning)	Prediction of lymph node metastasis from EBUS images	Support for nodal sampling decision-making during EBUS-TBNA	53 patients, 90 lymph nodes (balanced dataset; 3060+ image frames)	Very high diagnostic performance with SqueezeNet (accuracy, sensitivity, specificity, PPV, NPV all ≈ 96.7% using Adam optimizer); significantly outperformed SVM baseline; demonstrated feasibility of automated frame extraction from EBUS videos	Small sample size after selection, single-center retrospective design, no external validation, potential instability in training (Adam), and occasional mislocalization of relevant regions (Grad-CAM inconsistency)
Z. Chen et al. (2023) [30]	CNN (VGG19) + radiomics + clinical features (multimodal model)	Characterization of solitary pulmonary tumors using EBUS images	Complementary differential diagnosis integrating imaging and clinical data	EBUS dataset with manually selected ROIs (5-fold cross-validation)	Good diagnostic performance with multimodal fusion (AUC 85.14%, accuracy 80.55%, sensitivity 80.14%, specificity 81.69%, and F1-score 80.88%); performance improved with feature fusion and selection compared to single/dual modalities	Manual ROI delineation and clinician-dependent feature extraction (time-consuming), lack of full automation, and potential challenges for clinical integration of multimodal pipelines
I. F. Churchill et al. (2022) [31]	NeuralSeg (U-Net-based CNN segmentation + logistic regression)	Prediction of lymph node metastasis from EBUS images	Prioritization and risk stratification of suspicious lymph nodes during EBUS-TBNA	406 lymph nodes (298 derivation + 108 prospective validation)	High specificity (90.79%) and good negative predictive value (75.92%), supporting its role in ruling out malignancy; overall accuracy 72.87%; improved performance when combined with clinician scoring (accuracy 84.3% and NPV 90.22%)	Moderate sensitivity with notable false-negative rate, dependence on segmentation quality, and need for integration with clinical assessment to improve performance
C. K. Lin et al. (2021) [32]	CNN (ResNet101 for classification + HRNet for segmentation)	Analysis of lung cytology images during ROSE	Rapid cytological diagnosis and malignant cell localization during EBUS procedures	97 patients, 499 cytologic images (patch-based augmentation to 7486 patches)	Very high performance in patch-based classification (accuracy, sensitivity, and specificity all 98.8%); strong image-level accuracy (95.5%) and patient-level sensitivity (100%); effective segmentation with HRNet (mIoU 89.2%), enabling precise localization of malignant cells	Lower specificity at image/patient level, small test cohort, class imbalance handling required, and lack of prospective external validation
B. Khomkham et al. (2022) [33]	Ensemble (Random Forest + CNN + DenseNet169 multi-model framework)	Classification of pulmonary lesions (benign vs. malignant)	AI-assisted diagnosis using multimodal EBUS data (images + clinical features)	200 patients/200 EBUS images (124 malignant, 76 benign; augmented training set to 602 images)	High diagnostic performance with ensemble approach (accuracy 95%, sensitivity 100%, specificity 86.7%, and AUC 0.933); improved performance through integration of radiomics, clinical data, and image-based models	Small dataset with reliance on augmentation, limited generalizability, potential misclassification in small lesions (few patches), and no external validation
K. L. Yu et al. (2023) [34]	CNN (EfficientNet-B0) + test-time augmentation (TTA) + fine-tuning	Differentiation of benign vs. malignant lesions in rEBUS images	Automated interpretation of rEBUS images for diagnostic support	Multicenter retrospective study (3 centers; training: 260 patients; external cohorts: 190 and 35 lesions; >1100 images total)	Good performance in internal validation (AUC 0.88, sensitivity 0.85, and specificity 0.97); moderate performance in external validation (AUC 0.65–0.75), improved with TTA + fine-tuning (AUC up to 0.82 and accuracy ~0.79–0.80)	Reduced performance across centers, need for external fine-tuning, variable sensitivity, and limited generalizability
J. E. Oh et al. (2025) [35]	ResNet18 + multimodal integration (EBUS + ROI + CT + PET-CT)	Prediction of mediastinal lymph node metastasis	Multimodal integration for lung cancer staging and nodal assessment	Retrospective study (1454 patients; 2901 EBUS images from 2055 LN stations)	Excellent performance with multimodal model (AUROC 0.914, accuracy 82.3%, sensitivity 84.1%, and specificity 81.1%); significant improvement with PET-CT integration compared to EBUS alone (AUROC 0.870 → 0.914)	Limited sensitivity for false-negative nodes (21.4%), retrospective design, manual ROI annotation required, and high technical complexity for multimodal integration
T. Hotta et al. (2022) [36]	CNN (custom architecture)	Differentiation of benign vs. malignant peripheral pulmonary lesions	Diagnostic support in radial EBUS (EBUS-GS) procedures	Retrospective single-center study (213 patients; 171 lesions training, 42 lesions test; 2,421,360 augmented images; 26,674 test images)	Good overall performance (accuracy 83.4%); very high sensitivity (95.3%) with moderate NPV (82.0%); outperformed bronchoscopists in accuracy (83.3% vs. 68.5%)	Low specificity (53.4%) with high false-positive rate; single-center design; heavy reliance on data augmentation; limited lesion-level sample size despite large image dataset
C. W. Wang et al. (2022) [37]	Patch-based hierarchical CNN (modified FCN)	Segmentation of metastatic lymph node lesions in EBUS-TBNA cytology	Automated support for ROSE in whole-slide images (WSI)	Retrospective single-center study (122 WSIs from 62 patients; 47 malignant, 75 benign)	High segmentation performance: precision 93.4%, sensitivity 89.8%, Dice coefficient 82.2%, IoU 83.2%; significantly outperformed U-Net, SegNet, and FCN (*p* < 0.001); efficient WSI processing (<1 min per slide with multi-GPU)	Limited dataset size and single-center design; retrospective nature; limited diversity of cytological patterns; reliance on pixel-level annotations
J. Chen et al. (2025) [11]	AI-CEMA (multimodal deep learning with automatic frame selection and LN detection)	Diagnosis of benign vs. malignant intrathoracic lymphadenopathy	Fully automated analysis of Convex Probe-EBUS multimodal videos (B-mode, Doppler, and elastography) with representative frame selection	1006 LNs (training/retrospective, single center) + 267 LNs (prospective, multicenter)	Strong multimodal performance: AUC 0.889 (retrospective) and 0.849 (prospective); high sensitivity (97.1%) but moderate specificity (52.6%); comparable to expert performance; real-time capability (~23.7 FPS, ~40 ms latency)	Variable generalization across centers; low specificity and misclassification of benign diseases; threshold calibration required; high technical complexity
Y. S. Patel et al. (2024) [38]	Ensemble DNN (ResNet152V2 + InceptionV3 + DenseNet201 with MLP fusion)	Prediction of lymph node malignancy in NSCLC	AI-assisted nodal staging using EBUS-TBNA ultrasound images	2569 LN images from 773 patients (prospective dataset; 80/20 split)	Moderate overall performance: accuracy 80.6%, AUC 0.701; very high specificity (96.9%) and PPV (85.9%), but low sensitivity (43.2%); effective for confirming malignancy rather than screening	Low sensitivity limits detection of malignant cases; class imbalance; moderate AUC; requires larger datasets and further optimization before clinical adoption
H. Wang et al. (2025) [39]	M3-Net (multi-branch deep learning with attention-based feature fusion)	Diagnosis of lung cancer from EBUS images	Computer-aided diagnosis (CAD) system for peripheral lung lesions using EBUS-TBLB	95 patients (82 malignant, 13 benign); 1140 EBUS images extracted from videos	Moderate performance with best AUC ≈ 0.79 and improved results through multi-feature fusion (up to +8% AUC vs. single modality); performance enhanced using weighted loss and multi-scale inputs	Small and highly imbalanced dataset; single-center retrospective design; moderate overall performance
E. Amante et al. (2025) [40]	Deep learning (ResNet-50-based CNN on Iriscope video frames)	Prediction of malignancy in peripheral pulmonary nodules (rEBUS + Iriscope)	Decision-support tool for bronchoscopists, particularly less experienced operators	61 patients (PPL < 20 mm); 62,072 video frames	Moderate performance with balanced accuracy ~71.5%, sensitivity ~68% and specificity ~75% (optimal 45-frame window); outperformed junior physicians but remained inferior to experts; demonstrates value as an assistive tool	Small single-center retrospective cohort; limited sample size; variability depending on window size; performance inferior to expert bronchoscopists
Ø. Ervik et al. (2025) [41]	DNN (MobileNetV3 + LSTM for spatiotemporal analysis)	Automatic classification of thoracic lymph node stations	Real-time anatomical navigation support during EBUS-TBNA	28,134 EBUS images/56 patients	Moderate performance with accuracy 59.5% (stateful mode) vs. 54.6% (stateless); improved performance using temporal information; real-time feasibility demonstrated (≈0.65 s per prediction)	Small cohort; limited accuracy for clinical deployment; variability across lymph node stations; early exploratory stage
N. Ozcelik et al. (2020) [42]	ANN based on textural features (ROI and LN)	Differentiation of benign vs. malignant mediastinal lymph nodes	Diagnostic support using quantitative texture analysis in EBUS images	345 images (300 training/45 testing)	Acceptable performance: accuracy up to 85% (LN pattern) and AUC 0.782; lower performance in ROI (accuracy ~64%)	Small dataset, manual ROI segmentation, retrospective design, and limited generalizability

**Abbreviations:** AI: Artificial intelligence; ANN: artificial neural network; CNN: convolutional neural network; DNN: deep neural network; SVM: support vector machine; MLP: multilayer perceptron; KNN: k-nearest neighbors; LSTM: long short-term memory; Grad-CAM: gradient-weighted class activation mapping; EBUS: endobronchial ultrasound; EBUS-TBNA: endobronchial ultrasound-guided transbronchial needle aspiration; rEBUS: radial endobronchial ultrasound; EBUS-GS: EBUS with guide sheath; EBUS-TBLB: EBUS-guided transbronchial lung biopsy; ROSE: rapid on-site evaluation; ROI: region of interest; LN: lymph node; PET/CT: positron emission tomography/computed tomography; CT: computed tomography; CAD: computer-aided diagnosis; SAR: stiffness area ratio; CLNS: combined lymph node score; AUC/AUROC: area under the receiver operating characteristic curve; PPV: positive predictive value; NPV: negative predictive value; MCC: Matthews correlation coefficient; IoU: intersection over union; mIoU: mean intersection over union; FCN: fully convolutional network; WSI: whole slide image; TTA: test-time augmentation; NSCLC: non-small cell lung cancer; FPS: frames per second.

**Table 2:** Structured framework of heterogeneity in artificial intelligence applications for endobronchial ultrasound.

Dimension of Heterogeneity	Categories	Description	Representative Studies
Imaging modality	CP-EBUS	Convex probe EBUS for mediastinal lymph node evaluation and staging	[11,20,21,27,31,35]
	RP-EBUS	Radial probe EBUS for peripheral pulmonary lesions	[25,26,34,36,40]
	ROSE/Cytology	Rapid on-site cytological evaluation and whole-slide imaging	[28,32,37]
Input data type	Static images	Selected frames or regions of interest from EBUS	[20,22,27,29,42]
	Video-based	Full EBUS video sequences or temporal data	[13,25,40,41]
	Multimodal	Integration of EBUS with CT, PET-CT, or clinical variables	[11,24,30,33,35]
Validation strategy	Internal validation	Cross-validation or random split within same dataset	[22,25,30,39]
	External validation	Independent test datasets from different cohorts	[11,31,34]
	Multicenter validation	Data from multiple institutions	[11,34]
Clinical objective	Diagnosis	Benign vs. malignant lesion classification	[22,13,33,36,39]
	Staging	Lymph node metastasis prediction	[24,27,31,35,38]
	Navigation	Lymph node station classification/anatomical guidance	[21,41]
	Segmentation/Cytology	Lesion, lymph node or cell segmentation	[20,28,37]

**Abbreviations**: CP-EBUS: Convex probe endobronchial ultrasound; RP-EBUS: radial probe endobronchial ultrasound; ROSE: rapid on-site evaluation; CT: computed tomography; PET-CT: positron emission tomography–computed tomography.

## Data Availability

The datasets used in this study can be found in the full-text articles that were included in the scoping review.

## Supplementary Materials

The following supporting information can be downloaded at: https://www.mdpi.com/article/10.3390/curroncol33050287/s1, File S1: PRISMA Extension for Scoping Reviews (PRISMA-ScR) 2018 Checklist [17]; File S2: Search Strategies (search updated 1 January 2026).

## References

[1] Smolarz B., Łukasiewicz H., Samulak D., Piekarska E., Kołaciński R., Romanowicz H. (2025). Lung Cancer-Epidemiology, Pathogenesis, Treatment and Molecular Aspect (Review of Literature). Int. J. Mol. Sci..

[2] Deshpand R., Chandra M., Rauthan A. (2022). Evolving trends in lung cancer: Epidemiology, diagnosis, and management. Indian J. Cancer.

[3] Lynge E., Andersen B., Bojesen S.E., Petersen L.K., Bech M., Bøgsted M., Dam M.S., Egstrand S., Hvass A.M.F., Kobylecki C.J. (2026). Future of Cancer Screening Working Group. Challenges in the future of cancer screening. Int. J. Cancer.

[4] Bouchard N., Daaboul N. (2025). Lung Cancer: Targeted Therapy in 2025. Curr. Oncol..

[5] Lam S., Baldwin D.R., Devaraj A., Field J., Henschke C.I., Heuvelmans M.A., Huber R.M., Jones C., Borondy-Kitts A., Ching Li M.S. (2026). A Game-Changing 20 Years: Progress and Future Directions in Lung Cancer Screening. J. Thorac. Oncol..

[6] Qi Y.J., Zhang J., Salcedo Lobera E., Song Q.Y., Zhong R.H., Kontogianni K., Huang Z.S., Ariza-Prota M., Gupta N., Madan M. (2026). Endobronchial ultrasound-guided mediastinal biopsies for the diagnosis of mediastinal diseases: A network meta-analysis. Pulmonology.

[7] Fan Y., Zhang A.M., Wu X.L., Huang Z.S., Kontogianni K., Sun K., Fu W.L., Wu N., Kuebler W.M., Herth F.J.F. (2023). Transbronchial needle aspiration combined with cryobiopsy in the diagnosis of mediastinal diseases: A multicentre, open-label, randomised trial. Lancet Respir. Med..

[8] Tuta-Quintero E., Giraldo-Cadavid L.F., Sanmiguel-Reyes C., Navia M.E., Cardenas R., Bastidas A., Mora A., Páez-Espinel N., Viola L., Suárez M. (2025). Optimizing diagnostic yield in pulmonary lesions: Impact of combined sampling tools and EBUS-TBNA during radial EBUS. Ther. Adv. Respir. Dis..

[9] Ohannesian V.A., Falcão L., Ishizuka B.M., Menezes I.R., Han M.L., Suruagy-Motta R.F.O., Maximiano M.L.B., Cordeiro D.M.H., Baptista J.M., Mariussi M. (2025). The diagnostic accuracy of artificial intelligence models in detecting lymph node metastases in lung cancer using endobronchial ultrasound (EBUS) images: A bivariate meta-analysis. Clin. Imaging.

[10] Winiarski S., Radziszewski M., Wiśniewski M., Cisek J., Wąsowski D., Plewczyński D., Górska K., Korczyński P. (2025). Integrating Artificial Intelligence in Bronchoscopy and Endobronchial Ultrasound (EBUS) for Lung Cancer Diagnosis and Staging: A Comprehensive Review. Cancers.

[11] Chen J., Li J., Zhang C., Zhi X., Wang L., Zhang Q., Yu P., Tang F., Zha X., Wang L. (2025). Deep learning for detection and diagnosis of intrathoracic lymphadenopathy from endobronchial ultrasound multimodal videos: A multi-center study. Cell Rep. Med..

[12] Yong S.H., Lee S.H., Oh S.I., Keum J.S., Kim K.N., Park M.S., Chang Y.S., Kim E.Y. (2022). Malignant thoracic lymph node classification with deep convolutional neural networks on real-time endobronchial ultrasound (EBUS) images. Transl. Lung Cancer Res..

[13] Lin C.K., Wu S.H., Chua Y.W., Fan H.J., Cheng Y.C. (2025). TransEBUS: The interpretation of endobronchial ultrasound image using hybrid transformer for differentiating malignant and benign mediastinal lesions. J. Formos. Med. Assoc..

[14] Arksey H., O’Malley L. (2005). Scoping studies: Towards a methodological framework. Int. J. Soc. Res. Methodol..

[15] Levac D., Colquhoun H., O’Brien K.K. (2010). Scoping studies: Advancing the methodology. Implement. Sci..

[16] Aromataris E., Munn Z. (2020). JBI Manual for Evidence Synthesis.

[17] Tricco A.C., Lillie E., Zarin W., O’Brien K.K., Colquhoun H., Levac D., Moher D., Peters M.D.J., Horsley T., Weeks L. (2018). PRISMA Extension for Scoping Reviews (PRISMA-ScR): Checklist and Explanation. Ann. Intern. Med..

[18] Ouzzani M., Hammady H., Fedorowicz Z., Elmagarmid A. (2016). Rayyan—A web and mobile app for systematic reviews. Syst. Rev..

[19] Grudniewicz A., Nelson M., Kuluski K., Lui V., Cunningham H.V., XNie J., Colquhoun H., Wodchis W.P., Taylor S., Loganathan M. (2016). Treatment goal setting for complex patients: Protocol for a scoping review. BMJ Open.

[20] Ervik Ø., Tveten I., Hofstad E.F., Langø T., Leira H.O., Amundsen T., Sorger H. (2024). Automatic Segmentation of Mediastinal Lymph Nodes and Blood Vessels in Endobronchial Ultrasound (EBUS) Images Using Deep Learning. J. Imaging..

[21] Ervik Ø., Rødde M., Hofstad E.F., Langø T., Leira H.O., Amundsen T., Sorger H. (2026). Explainability of a Deep Learning Model for Mediastinal Lymph Node Station Classification in Endobronchial Ultrasound (EBUS). Bioengineering.

[22] Chen C.H., Lee Y.W., Huang Y.S., Lan W.R., Chang R.F., Tu C.Y., Chen C.Y., Liao W.C. (2019). Computer-aided diagnosis of endobronchial ultrasound images using convolutional neural network. Comput. Methods Programs Biomed..

[23] Patel Y.S., Gatti A.A., Farrokhyar F., Xie F., Hanna W.C. (2024). Clinical utility of artificial intelligence-augmented endobronchial ultrasound elastography in lymph node staging for lung cancer. JTCVS Tech..

[24] Guberina M., Herrmann K., Pöttgen C., Guberina N., Hautzel H., Gauler T., Ploenes T., Umutlu L., Wetter A., Theegarten D. (2022). Prediction of malignant lymph nodes in NSCLC by machine-learning classifiers using EBUS-TBNA and PET/CT. Sci. Rep..

[25] Wang H., Nakajima T., Shikano K., Nomura Y., Nakaguchi T. (2025). Classification of peripheral pulmonary lesions in Endobronchial ultrasonography image using a multi-branch framework and voting ensemble. Comput. Biol. Med..

[26] Xing J., Li C., Wu P., Cai X., Ouyang J. (2024). Optimized fuzzy K-nearest neighbor approach for accurate lung cancer prediction based on radial endobronchial ultrasonography. Comput. Biol. Med..

[27] Ito Y., Nakajima T., Inage T., Otsuka T., Sata Y., Tanaka K., Sakairi Y., Suzuki H., Yoshino I. (2022). Prediction of Nodal Metastasis in Lung Cancer Using Deep Learning of Endobronchial Ultrasound Images. Cancers.

[28] Lan H., Chen P., Wang C., Chen C., Yao C., Jin F., Wan T., Lv X., Wang J. (2024). A Multiscale Connected UNet for the Segmentation of Lung Cancer Cells in Pathology Sections Stained Using Rapid On-Site Cytopathological Evaluation. Am. J. Pathol..

[29] Ishiwata T., Inage T., Aragaki M., Gregor A., Chen Z., Bernards N., Kafi K., Yasufuku K. (2024). Deep learning-based prediction of nodal metastasis in lung cancer using endobronchial ultrasound. JTCVS Tech..

[30] Chen Z., Feng J., Wei X., He Q., Li S., Zhong C., Luo J. Multi-modal features for intelligent differential diagnosis of solitary pulmonary tumors by using endobronchial ultrasonography images. Proceedings of the 2023 IEEE International Ultrasonics Symposium (IUS).

[31] Churchill I.F., Gatti A.A., Hylton D.A., Sullivan K.A., Patel Y.S., Leontiadis G.I., Farrokhyar F., Hanna W.C. (2022). An Artificial Intelligence Algorithm to Predict Nodal Metastasis in Lung Cancer. Ann. Thorac. Surg..

[32] Lin C.K., Chang J., Huang C.C., Wen Y.F., Ho C.C., Cheng Y.C. (2021). Effectiveness of convolutional neural networks in the interpretation of pulmonary cytologic images in endobronchial ultrasound procedures. Cancer Med..

[33] Khomkham B., Lipikorn R. (2022). Pulmonary Lesion Classification Framework Using the Weighted Ensemble Classification with Random Forest and CNN Models for EBUS Images. Diagnostics.

[34] Yu K.L., Tseng Y.S., Yang H.C., Liu C.J., Kuo P.C., Lee M.R., Huang C.T., Kuo L.C., Wang J.Y., Ho C.C. (2023). Deep learning with test-time augmentation for radial endobronchial ultrasound image differentiation: A multicentre verification study. BMJ Open Respir. Res..

[35] Oh J.E., Chung H.S., Gwon H.R., Park E.Y., Kim H.Y., Lee G.K., Kim T.S., Hwangbo B. (2025). Prediction of Lymph Node Metastasis in Lung Cancer Using Deep Learning of Endobronchial Ultrasound Images With Size on CT and PET-CT Findings. Respirology.

[36] Hotta T., Kurimoto N., Shiratsuki Y., Amano Y., Hamaguchi M., Tanino A., Tsubata Y., Isobe T. (2022). Deep learning-based diagnosis from endobronchial ultrasonography images of pulmonary lesions. Sci. Rep..

[37] Wang C.W., Khalil M.A., Lin Y.J., Lee Y.C., Huang T.W., Chao T.K. (2022). Deep Learning Using Endobronchial-Ultrasound-Guided Transbronchial Needle Aspiration Image to Improve the Overall Diagnostic Yield of Sampling Mediastinal Lymphadenopathy. Diagnostics.

[38] Patel Y.S., Gatti A.A., Farrokhyar F., Xie F., Hanna W.C. (2024). Artificial Intelligence Algorithm Can Predict Lymph Node Malignancy from Endobronchial Ultrasound Transbronchial Needle Aspiration Images for Non-Small Cell Lung Cancer. Respiration.

[39] Wang H., Nakajima T., Shikano K., Nomura Y., Nakaguchi T. (2025). Diagnosis of Lung Cancer Using Endobronchial Ultrasonography Image Based on Multi-Scale Image and Multi-Feature Fusion Framework. Tomography.

[40] Amante E., Ghyselinck R., Thiberville L., Trisolini R., Guisier F., Delchevalerie V., Dumas B., Frénay B., Duparc I., Mazellier N. (2025). Human and Deep Learning Predictions of Peripheral Lung Cancer Using a 1.3 mm Video Endoscopic Probe. Respirology.

[41] Ervik Ø., Rødde M., Hofstad E.F., Tveten I., Langø T., Leira H.O., Amundsen T., Sorger H. (2025). A New Deep Learning-Based Method for Automated Identification of Thoracic Lymph Node Stations in Endobronchial Ultrasound (EBUS): A Proof-of-Concept Study. J. Imaging.

[42] Ozcelik N., Ozcelik A.E., Bulbul Y., Oztuna F., Ozlu T. (2020). Can artificial intelligence distinguish between malignant and benign mediastinal lymph nodes using sonographic features on EBUS images?. Curr. Med. Res. Opin..

[43] Tang F., Zha X.K., Ye W., Wang Y.M., Wu Y.F., Wang L.N., Lyu L.P., Lyu X.M. (2025). Artificial intelligence-assisted endobronchial ultrasound for differentiating between benign and malignant thoracic lymph nodes: A meta-analysis. BMC Pulm. Med..

[44] Cold K.M., Vamadevan A., Laursen C.B., Bjerrum F., Singh S., Konge L. (2025). Artificial intelligence in bronchoscopy: A systematic review. Eur. Respir. Rev..

[45] Petrick N., Chen W., Delfino J.G., Gallas B.D., Kang Y., Krainak D., Sahiner B., Samala R.K. (2023). Regulatory considerations for medical imaging AI/ML devices in the United States: Concepts and challenges. J. Med. Imaging.

[46] Suleman M.U., Mursaleen M., Khalil U., Saboor A., Bilal M., Khan S.A., Subhani M.A., Hussnain M.A., Tabassum S.N., Tahir M. (2025). Assessing the generalizability of artificial intelligence in radiology: A systematic review of performance across different clinical settings. Ann. Med. Surg..

[47] Matthews G.A., McGenity C., Bansal D., Treanor D. (2024). Public evidence on AI products for digital pathology. npj Digit. Med..

[48] Ogut E. (2025). Artificial Intelligence in Clinical Medicine: Challenges Across Diagnostic Imaging, Clinical Decision Support, Surgery, Pathology, and Drug Discovery. Clin. Pract..

[49] Echeverri-Hoyos J., Echeverri-Franco J.A., Bonilla N., Monsalve-Morales G., Quintero-Tuta E. (2026). Applications of Artificial Intelligence in Endobronchial Ultra-Sound for Lung Cancer Diagnosis and Staging: A Scoping Review.

[50] Collins G.S., Moons K.G.M., Dhiman P., Riley R.D., Beam A.L., Van Calster B., Ghassemi M., Liu X., Reitsma J.B., van Smeden M. (2024). TRIPOD+AI statement: Updated guidance for reporting clinical prediction models that use regression or machine learning methods. BMJ.

[51] Liu X., Cruz Rivera S., Moher D., Calvert M.J., Denniston A.K., Grupo de Trabajo SPIRIT-AI y CONSORT-AI (2024). Directrices para presentación de informes de ensayos clínicos sobre intervenciones con inteligencia artificial: Extensión CONSORT-AI. Rev. Panam. Salud Publica.

[52] Mongan J., Moy L., Kahn C.E. (2020). Checklist for Artificial Intelligence in Medical Imaging (CLAIM): A Guide for Authors and Reviewers. Radiol. Artif. Intell..

