# Supplementary material for: Applications of Artificial Intelligence in Endobronchial Ultrasound for Lung Cancer Diagnosis and Staging: A Scoping Review
Source: Curr Oncol. 2026 May 13;33(5):287. doi: 10.3390/curroncol33050287 (PMC13205784; doi:10.3390/curroncol33050287)
Supplement: Supplementary file 1 [file curroncol-33-00287-s001.zip › curroncol-4270730-supplementary.pdf]

Supplementary File S1. PRISMA Extension for Scoping Reviews (PRISMA-ScR) 2018 Checklist

Section/topic	#	PRISMA-ScR Checklist item	Reported on page #
**TITLE**			
Title	1	Identify the report as a scoping review.	1
**ABSTRACT**			
Structured summary	2	Provide a structured summary including, as applicable: background; objectives; data sources; study eligibility criteria, participants, and interventions; study synthesis methods; results; limitations; conclusions and implications of key findings.	2
**INTRODUCTION**			
Rationale	3	Describe the rationale for the review in the context of what is already known. Explain why the review question(s)/objective(s) lend themselves to a scoping review approach.	3
Objectives	4	Provide an explicit statement of the question(s) and objective(s) being addressed with reference to their key elements (e.g., population or participants, concepts and context), or other relevant key elements used to conceptualize the review question(s) and/or objective(s)).	3
**METHODS**			
Protocol and registration	5	Indicate if a review protocol exists, if and where it can be accessed (e.g., Web address), and, if available, provide registration information including registration number.	4
Eligibility criteria	6	Specify the characteristics of the sources of evidence (e.g., years considered, language, publication status) used as criteria for eligibility, and provide a rationale.	4
Information sources	7	Describe all information sources (e.g., databases with dates of coverage, contact with study authors to identify additional sources) in the search and date last searched.	5
Search	8	Present full electronic search strategy for at least one database, including any limits used, such that it could be repeated.	Supplementary file
Selection of sources of evidence	9	State the process for selecting studies (i.e., screening, eligibility) included in the scoping review.	4
Data charting process	10	Describe the methods of charting data from the included sources of evidence (e.g. piloted forms; forms that have been tested by the team before their use, whether data charting was done independently, in duplicate) and any processes for obtaining and confirming data from investigators.	4
Data items	11	List and define all variables for which data were sought and any assumptions and simplifications made.	5
Critical appraisal of individual	12	**If done,** provide a rationale for conducting a critical appraisal of included sources of evidence; describe the methods used and how	NA

Section/topic	#	PRISMA-ScR Checklist item	Reported on page #
sources of evidence		this information was used in any data synthesis (if appropriate).	
Summary measures	13	Not applicable for scoping reviews.	NA
Synthesis of results	14	Describe the methods of handling and summarizing the data that were charted.	4-5
Risk of bias across studies	15	Not applicable for scoping reviews.	NA
Additional analyses	16	Not applicable for scoping reviews.	NA
**RESULTS**			
Selection of sources of evidence	17	Give numbers of studies screened, assessed for eligibility, and included in the review, with reasons for exclusions at each stage, ideally using a flow diagram.	Figure 1
Characteristics of sources of evidence	18	For each source of evidence, present characteristics for which data were charted and provide the citations.	Table 1
Critical appraisal within sources of evidence	19	If done, present data on critical appraisal of included sources of evidence (see item 12).	NA
Results of individual sources of evidence	20	For each included source of evidence, present the relevant data that were charted that relate to the review question(s) and objective(s).	Table 1
Synthesis of results	21	Summarize and/or present the charting results as they relate to the review question(s) and objective(s).	6
Risk of bias across studies	22	Not applicable for scoping reviews.	NA
Additional analysis	23	Not applicable for scoping reviews.	NA
**DISCUSSION**			
Summary of evidence	24	Summarize the main results (including an overview of concepts, themes, and types of evidence available), explain how they relate to the review question(s) and objectives, and consider the relevance to key groups	7-8
Limitations	25	Discuss the limitations of the scoping review process.	7-8
Conclusions	26	Provide a general interpretation of the results with respect to the review question(s) and objective(s), as well as potential implications and/or next steps.	7-8
**FUNDING**			
Funding	27	Describe sources of funding for the included sources of evidence, as well as sources of funding for the scoping review. Describe the role	NA

Section/topic	#	PRISMA-ScR Checklist item	Reported on page #
		of the funders of the scoping review.	

Tricco AC, Lillie E, Zarin W, et al. PRISMA Extension for Scoping Reviews (PRISMA-ScR): Checklist and Explanation. *Ann Intern Med* 2018;169(7):467-473.

Supplementary File S2. Search Strategies (search updated 1 January 2026)

PubMed/MEDLINE 70
PubMed - ("Artificial Intelligence" OR AI OR "Machine Learning" OR "Deep Learning" OR "Neural Networks" OR "Computer-aided diagnosis" OR CAD) AND ("Lung cancer" OR "Pulmonary carcinoma" OR NSCLC OR SCLC) AND ("Endobronchial ultrasound" OR EBUS OR "EBUS-TBNA" OR "Bronchoscopic ultrasound")
Scopus 57
Scopus - TITLE-ABS-KEY (("Artificial Intelligence" OR AI OR "Machine Learning" OR "Deep Learning" OR "Neural Network*" OR "Computer-aided diagnosis" OR CAD) AND ("Lung cancer" OR "Pulmonary carcinoma" OR NSCLC OR SCLC) AND ("Endobronchial ultrasound" OR EBUS OR "EBUS-TBNA" OR "Bronchoscopic ultrasound"))
Embase 143
Embase - ('endobronchial ultrasound'/exp OR ebus OR 'ebus-tbna' OR 'endobronchial ultrasonography') AND ('artificial intelligence'/exp OR 'machine learning'/exp OR 'deep learning'/exp OR 'neural network'/exp OR 'computer assisted diagnosis'/exp OR 'image processing'/exp OR 'pattern recognition'/exp OR 'radiomics'/exp OR 'artificial intelligence' OR 'machine learning' OR 'deep learning' OR 'neural network*' OR radiomics OR 'image analysis' OR classification OR prediction) AND ('lung cancer'/exp OR 'lung tumor'/exp OR 'lung neoplasm'/exp OR 'pulmonary neoplasm*' OR cancer OR neoplasm* OR tumor* OR tumour*)
